# Dissimilar Gas Tungsten Arc Welding of (FeCoNi)_96_Al_4_ High-Entropy Alloy and Q235 Structural Steel

**DOI:** 10.3390/ma18020280

**Published:** 2025-01-10

**Authors:** Zhen Yang, Guorui Sun, Chao Chen

**Affiliations:** 1 College of Mechanical Engineering and Automation, Dalian Polytechnic University, Dalian 116034, China; 2 Key Laboratory of Automobile Materials, School of Materials Science and Engineering, Jilin University, Changchun 130025, China; sungr24@mails.jlu.edu.cn

**Keywords:** gas tungsten arc welding, high-entropy alloy, structural steels, microstructure, mechanical properties

## Abstract

(FeCoNi)_96_A_l4_ high-entropy alloy (HEA) is a new material with a strength similar to that of commercial Q235 structural steel, and its elongation is nearly three times greater than that of Q235 steel. Studying the welding process of the (FeCoNi)_96_Al_4_ HEA and Q235 steel is expected to further expand the application range of commercial Q235 structural steel and provide a foundation for the engineering application of the (FeCoNi)_96_Al_4_ HEA. This study focuses on the dissimilar welded components of (FeCoNi)_96_Al_4_ HEA and Q235 steel and analyzes the forming quality, microstructure, and mechanical properties of dissimilar welded samples under different currents. The results show that when the welding current is above 170 A, the 3 mm sheet metal is completely penetrated, and a well-formed weld seam is obtained. The base metal of the (FeCoNi)_96_Al_4_ HEA has an FCC structure, whereas the fusion zone of the weld seam is almost entirely a BCC structure. The microstructure of the weld seam exhibits needle-like and block-like grains that are different from those of the base metal. Owing to the difference in microstructure between the weld seam and the base metal, the average microhardness of the welded joint is twice that of the base metal. The strength of the dissimilar welded components reached 460 MPa, maintaining the tensile strength of the (FeCoNi)_96_Al_4_ HEA, which is similar to that of the Q235 structural steel. The elongation reached over 30%, which was significantly greater than that of the Q235 structural steel.

## 1. Introduction

High-entropy alloys (HEAs) are metal materials with high mixing entropy and excellent mechanical properties, corrosion resistance, high-temperature performance, and good processing performance [1,2,3]. Due to their unique properties, HEAs have broad application prospects, such as aerospace engines, nuclear power components, and key components in automotive body structures. HEAs are usually prepared and processed by melting, casting, welding, machining, and heat treatments [4,5,6]. The welding process of HEAs is one of the key steps in preparing complex HEA components and is also the key to their application in engineering fields [2,7].

Researchers have extensively researched the welding of HEAs. The welding methods commonly used are gas tungsten arc welding [8,9,10,11], gas metal arc welding [12], laser welding [13,14,15], and electron beam welding [2,16]. Wu et al. [16] studied the welding performance of custom-made CrMnFeCoNi HEA thin plates via electron beam welding. After welding, the ductility of the substrate is maintained. The weld quality, including weld formation, surface condition, and internal defect condition, and the mechanical properties are good, without obvious defects, verifying the room-temperature weldability of CrMnFeCoNi. Oliveira et al. [8] studied the GTAW (Gas Tungsten Arc Welding) of CrMnFeCoNi HEA rolled sheets. By characterizing the microstructure of the joint, it was found that the grain size in the fusion zone was large. Moreover, the hardness of this area is relatively low. The tensile test results indicate that fractures occur in the fusion zone of the weld seam. Kashaev et al. [17] used lasers to weld a CrMnFeCoNi HEA. The results indicate that the presence of M7C3-type carbides in the weld seam significantly increases the hardness and stabilizes the mechanical properties of each joint, and fractures occur at the base metal location. In addition, scholars have also studied the friction stir welding (FSW) of HEAs. Park et al. [18] conducted FSW on rolled and cast HEAs. The rolled HEA joint fractures in the fusion zone due to thinning, whereas the cast HEA joint fractures in the base metal area. The low-temperature applicability of HEA joints was verified through mechanical performance testing.

HEA, as a new type of metal material, can have wider application prospects and a more important value if it is welded with other metal materials. Further in-depth research is needed on the welding process, performance optimization, and applicability of HEAs to other metal materials in various fields to promote the further development of HEAs.

Dissimilar welding between HEAs and steels is an important welding technique. Owing to the significant difference in chemical composition between HEAs and steels, defects such as brittle phases and cracks are prone to occur during the welding process [14,19]. However, through appropriate welding methods and process parameter optimization, heterogeneous welding of HEAs and steels still achieves good results.

Oliveira et al. [13] developed defect-free CoCrFeMnNi and 316 structural steel joints via laser welding. A new solid solution formed in the weld seam. The hardness in the weld fusion zone increases, and a fracture also occurs in the fusion zone. Xin et al. [14] studied the weld seams of CrMnFeCoNi and 316LN structural steel. The results indicate that deformation substructures, which are composed of dislocations, faults, and nanotwins, appear in the fusion zone. The joint breaks in the fusion zone. Oliveira et al. [15] used laser welding to weld annealed CoCrFeMnNi HEA and 316 structural steel. Compared with that of the rolled CoCrFeMnNi sample, the strength of the annealed sample did not significantly decrease, whereas the ductility significantly improved. Sokkalingam et al. [2]. conducted dissimilar welding of Al_0.1_CoCrFeNi and AISI 304. A complete austenite microstructure was formed in the weld. Tensile testing indicated that the welded material exhibited a greater strength than the Al_0.1_CoCrFeNi HEA. The reliability of using electron beam welding for the welding of an Al_0.1_CoCrFeNi alloy was verified.

Although the feasibility of heterogeneous welding for HEAs has been proven, research on the heterogeneous welding for HEAs is also increasing. However, the heterogeneous welding of HEAs faces many challenges. First, the high melting point of HEAs makes the welding process more difficult. Second, various elements in HEAs have different chemical reactivities, which can easily lead to the formation of pores and cracks during the welding process [20,21,22,23,24]. In addition, as scholars have further expanded their research on HEAs, the types of HEAs are also constantly increasing. To provide more options for HEAs in the engineering field, further research is needed on the welding of HEA materials and dissimilar welding processes. The weldability of (FeCoNi)_96_Al_4_ alloys was investigated in our previous work, and in this study, (FeCoNi)_96_Al_4_ was joined with structural steel dissimilarities to further explore the value of (FeCoNi)_96_Al_4_ for engineering applications. Welding small amounts of (FeCoNi)_96_Al_4_ HEA to Q235 steel gives structural steel a higher service life in applications where material properties are more demanding.

As a new material, the tensile strength of the (FeCoNi)_96_Al_4_ HEA is similar to that of commercial Q235 structural steel, but its elongation is much greater than that of commercial Q235 steel. Although HEA contains expensive elements such as cobalt, nickel, and aluminum, it can be used in welded structures in small quantities with Q235 steel, which can be utilized in load-bearing, volatile areas to significantly improve overall performance and enable efficient use of expensive materials. Owing to the excellent corrosion resistance and mechanical properties of HEA materials, in this work, we conducted dissimilar welding of (FeCoNi)_96_Al_4_ HEA materials with commercial Q235 carbon structural steel. The forming quality, microstructure, and mechanical properties of the welds under different welding currents were analyzed, and the dissimilar welding process of the (FeCoNi)_96_Al_4_ HEA was studied. We explore whether the (FeCoNi)_96_Al_4_ HEA can be applied in commercial Q235 structural steel usage scenarios, providing choices for the (FeCoNi)_96_Al_4_ HEA and Q235 carbon structural steel to be used on more occasions.

## 2. Experimental Methods

In this study, the welding plate used is commercial Q235 carbon structural steel. The chemical composition of the (FeCoNi)_96_Al_4_ HEA plates (BEIJING RYUBON NEW MATERIAL TECHNOLOGY CO., Ltd., Beijing, China), prepared by the vacuum suspension melting technique, is shown in Table 1. The Q235 structural steel and (FeCoNi)_96_Al_4_ HEA are cut into 99 × 55 × 3 mm^3^ thin plates via CNC cutting machines. Before welding, mechanical polishing is used to remove oxide films and other pollutants from the mating surfaces and the surfaces of the plates.

The GTAW welding power source selected for the experiment is the KEMPPI brand MasterTig 335AC/DC high-frequency welding machine (Kemppi, Lahti, Finland), and the current mode is DC. After preliminary experimental verification, welding currents of 160 A, 170 A, 180 A, and 190 A were selected, and the welding samples were recorded as W1, W2, W3, and W4, respectively. The tungsten electrode was fixed at a position 4 mm above the plate to be welded, and a welding speed of 5 mm/s was chosen. The protective gas selected was 99.999% pure argon gas (15 L/min). The welding heat input is calculated via the formula Q = UI/V, where U is the welding voltage, I is the welding current, and V is the welding speed. The heat inputs of joints W1, W2, W3, and W4 are 524.8, 557.6, 590.4, and 623.2 J/mm, respectively.

After the welding process was completed, to analyze the quality of weld surface formation, a digital photography platform was used to take photos of the weld surface. A precision wire cutting machine was used to cut samples for microstructure characterization. The microstructure analysis sample was polished via standard metallographic sample preparation methods. Owing to the lower corrosion resistance of the Q235 carbon structural steel compared to that of the (FeCoNi)_96_Al_4_ HEA, aqua regia cannot be used for the corrosion of metallographic samples. A corrosion solution ratio of 10% HNO_3_ and 90% H_2_O was chosen. After 15 s of corrosion, the samples were quickly rinsed and dried. After the corrosion process, an Axio Vert A1 optical microscope and a Hitachi SU500 scanning electron microscope (SEM) were used for a microscopic morphology analysis. Energy dispersive spectroscopy (EDS) is used to observe the distribution of elements in and around welds. An INCACrystal Electron Backscatter Diffraction (EBSD) instrument (Oxford Instruments, Oxford, UK) was used to collect crystallographic information. Before the EBSD test, the sample was electropolished in a solution of 90 vol% alcohol + 10 vol% perchloric acid.

The tensile sample was cut at a stable position on the weld seam. The tensile specimen is perpendicular to the weld, and the test area contains both the weld and the base metal. The tensile test was conducted using a WDW-100 testing machine (Jinan Yanrui Testing Instrument Co., Ltd., Jinan, China) at room temperature. To ensure the credibility of the test data, the tensile test was repeated three times. Hardness tests were performed via an HVS-1000 hardness tester (200 g load) with a point spacing of 40 μm.

## 3. Results and Discussion

**Figure 1:**
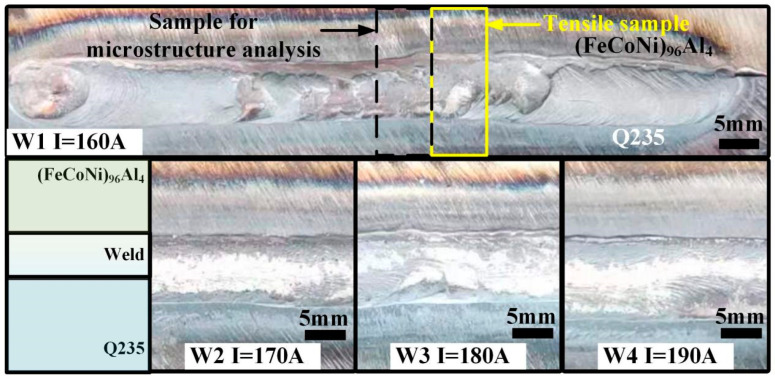
Micrographs of the weld with 160–190 A welding current.

Figure 1 shows the macroscopic diagram of the weld seam at a welding current of 160–190 A. The welding current is 160 A, and the heat input in the early stage of welding is small. In the first half of the weld seam, the melting amount of the Q235 structural steel plate is large, and it is slightly fused with the (FeCoNi)_96_Al_4_ HEA plate. After a certain amount of heat accumulates in the second half of the weld seam, the two types of plates tend to melt steadily and merge symmetrically. However, during this heat input, a small amount of the nonfusion zone appears at the bottom of the sheet. When the welding current increases to 170 A or above (heat input ≥ 557.6 J/mm), the two types of plates in the first half of the weld are uniformly fused, and there is no incomplete fusion area at the bottom of the plate, resulting in a fully penetrated and well-formed weld.

**Figure 2:**
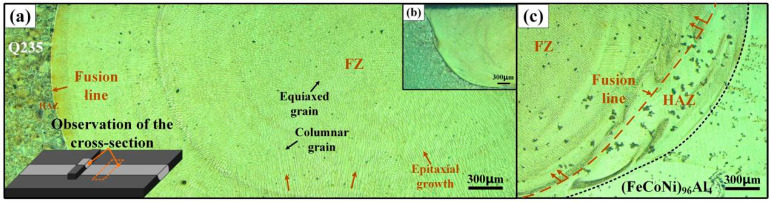
Microstructure at different positions of the weld seam with 170 A and 180 A welding currents. (**a**) 180 A current Q235 fusion line position, (**b**) 170 A current Q235 fusion line position, (**c**) 180 A current (FeCoNi)_96_Al_4_ fusion line position. Fusion zone (FZ), heat-affected zone (HAZ).

Figure 2 shows the microstructure of the weld seam at 170 and 180 A welding currents. The overall macroscopic weld seam presents a cup-shaped, asymmetric structure. The microstructure of the Q235 steel plate is composed of ferrite and pearlite. After being etched with a 4% nitric acid solution, a white ferrite matrix phase and black pearlite phase formed, as shown in Figure 2a. After arc heating, some grains in the HAZ grow due to overheating, and needle-like ferrite appears in the coarse pearlite grains. Near the fusion line, the needle-like structure grows in a direction perpendicular to the fusion line. Owing to the overheating of the steel during cooling, the first eutectoid ferrite precipitates in a needle-like manner along a certain crystal plane. At the bottom and edge of the molten pool, many columnar grains are generated and grow toward the center of the molten pool in the form of epitaxial crystallization, which is due to the large temperature gradient. At the center of the molten pool, the temperature gradient is small, and heat is transmitted in all directions, resulting in equiaxed grain growth. At the fusion line of (FeCoNi)_96_Al_4_, owing to the high welding heat, there is a large area of heat-affected zone, and an obvious partial melting zone can also be observed, as shown in Figure 2c.

**Figure 3:**
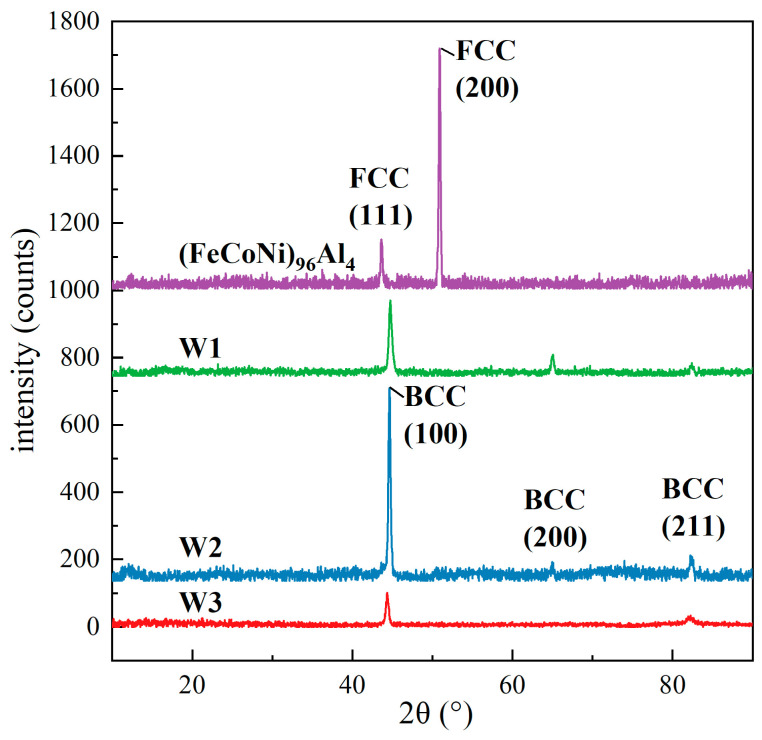
X-ray diffraction (XRD) patterns of the welded joints and (FeCoNi)_96_Al_4_ HEA base material.

Figure 3 shows the XRD patterns of the (FeCoNi)_96_Al_4_ alloy base material and weld seam. The detection results of the (FeCoNi)_96_Al_4_ HEA prepared via vacuum suspension melting technology indicate that it is composed of a single FCC phase. This detection result is similar to that of equiatomic CoCrFeMnNi HEA materials prepared via traditional processing routes [25,26]. In addition, small amounts of oxides and inclusions may exist during the alloy production process because of possible contamination. Q235 carbon structural steel is a typical ferritic steel that is mainly composed of ferrite, which may contain very small amounts of residual austenite and bainite. Its main ferrite phase is the typical BCC crystal structure [27,28]. According to the X-ray diffraction results of the welded joint area, only the BCC phase was indexed. The detection was conducted on W1, W2, and W3 welded joints. The results showed that only the BCC phase can be indexed in the dissimilar welds of the (FeCoNi)_96_Al_4_ HEA and the Q235 carbon structural steel. Although the (FeCoNi)_96_Al_4_ HEA is mainly composed of the FCC phase, only the BCC phase can be indexed to the melting zone. The mixing of various elements in the fusion zone (FZ) may change the overall chemical composition of the weld, and the original solidification path and phase composition may also be altered as a result.

**Figure 4:**
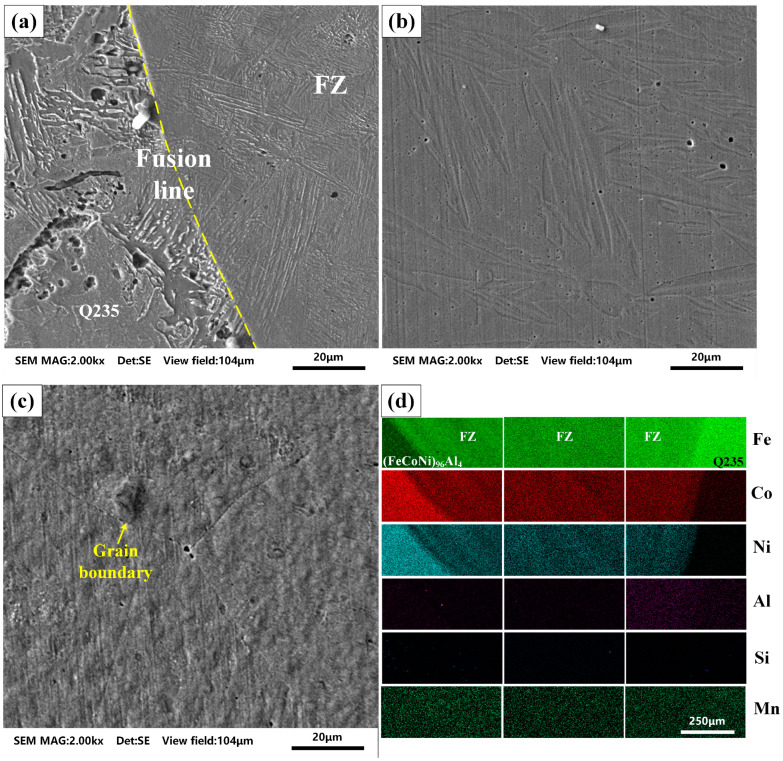
SEM images at different positions and distribution maps of major elements. (**a**) Q235 fusion line position, (**b**) weld center position, (**c**) (FeCoNi)_96_Al_4_ base metal, (**d**) element distribution diagram.

Figure 4 shows the microstructure of the cross section of the dissimilar weld at 170 A. The SEM image shows no significant microdefects near the central area of the weld, with only a small number of pores less than 3 μm observed. Q235 has a clear welding fusion line on one side. After being corroded by nitric acid solution, the black matrix exhibited a needle-like and layered morphology. No obvious fusion line was observed on the side of (FeCoNi)_96_Al_4_ in the SEM image. The SEM image of the (FeCoNi)_96_Al_4_ base material is shown in Figure 4c, with large corroded grain boundaries appearing in the base material. In arc welding technology, the width of the fusion zone is large, allowing grain growth to form slender or coarser columnar crystals. To analyze the solidification path of the average composition of the two substrates, the main elements (Fe, Co, Ni, Al, Si, and Mn) in the (FeCoNi)_96_Al_4_ HEA and Q235 steel were detected via an energy spectrometer, as shown in Figure 4d. Two types of substrate elements are mixed in the weld seam. The distribution of various elements in the weld seam is uniform, and there is no obvious segregation phenomenon. By observing the entire fusion zone of the weld, it was found that the main components are relatively evenly distributed in the weld.

**Figure 5:**
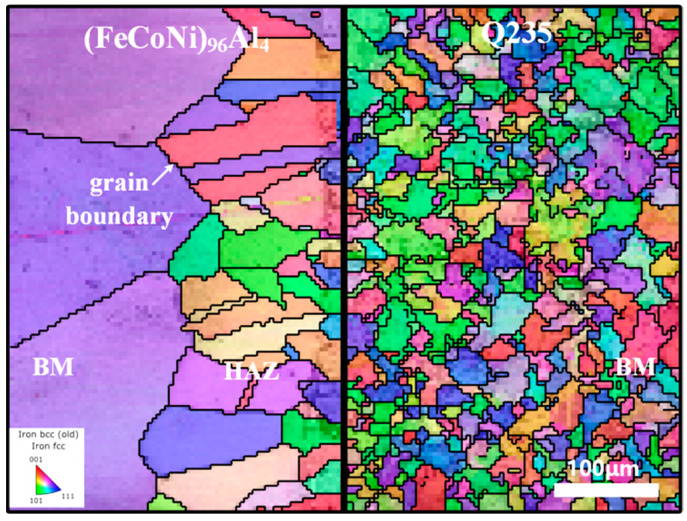
IPF map of the unfused area at the bottom of the melt pool with grain boundaries.

Figure 5 shows the inverse pole figure (IPF) map of the unfused area at the bottom of the melt pool with grain boundaries. This area displays IPF maps of the (FeCoNi)_96_Al_4_ and Q235 base materials, as well as IPF maps of the heat-affected zone. Commercial Q235 structural steel has a typical BCC crystal structure, whereas (FeCoNi)_96_Al_4_HEA was prepared via vacuum suspension melting technology and has an FCC crystal structure. Large-scale microstructure features can be observed in the base material, which is consistent with the results observed in the SEM image in Figure 4c. When the welding current is low, the welded plate fails to penetrate, and a small area of incomplete fusion appears at the bottom of the melt pool. However, owing to the influence of heat conduction in the arc hot melt pool, a clear welding heat-affected zone can be observed in this area. This area is similar to the HAZ on both sides of the weld. Grain recrystallization occurs due to welding thermal cycling. Through the heat conduction of the melt pool and the welding thermal cycle, the local temperature of the base metal rapidly increases, thereby promoting renucleation and nucleation growth inside the alloy, namely, recrystallization and grain growth. Through measurements, it was found that the HAZ width of a fully penetrated joint can reach over 300 μm.

**Figure 6:**
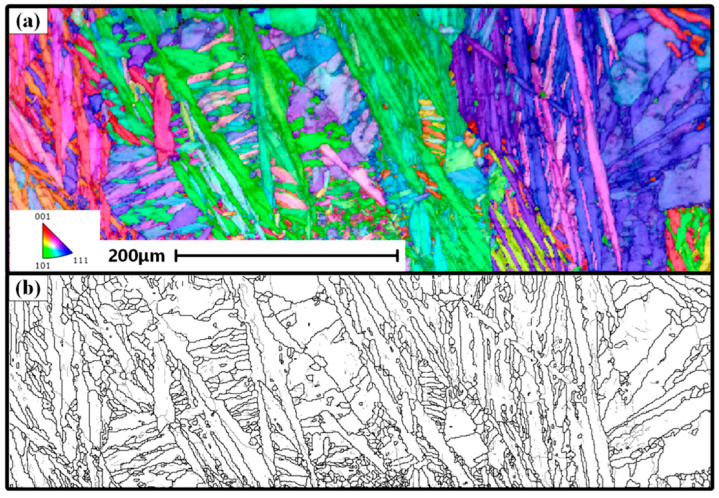
IPF map and grain boundary map at the center of the weld seam at a welding current of 170 A. (**a**) IPF map (**b**) Grain boundary map.

Figure 6 shows the IPF map at the center of the weld seam and the corresponding grain boundary map at a welding current of 170 A. According to Figure 2a, the BM and HAZ of the Q235 carbon structural steel are both typical low-carbon steel metallographic structures [29], with a typical BCC crystal structure. On the basis of the XRD results in Figure 3, the EBSD results also indicate the presence of over 99.5% of the BCC phase in the weld. However, the microstructure in the weld presents elongated, needle-like and small, piece-like structures. For low-carbon steel, the large temperature gradient of the weld after welding is also effective for the formation of a slender needle-like BCC phase in the fusion zone of the weld, as shown in Figure 6a. Owing to the complete incoherence within the grains, the BCC structure grows in one direction within the fusion zone of the weld, forming a needle-like structure. The EBSD analysis results further confirmed the presence of a needle-like BCC phase in the optical image. Moreover, the length of some needle-like structures in the fusion zone of the weld exceeds 300 μm.

**Figure 7:**
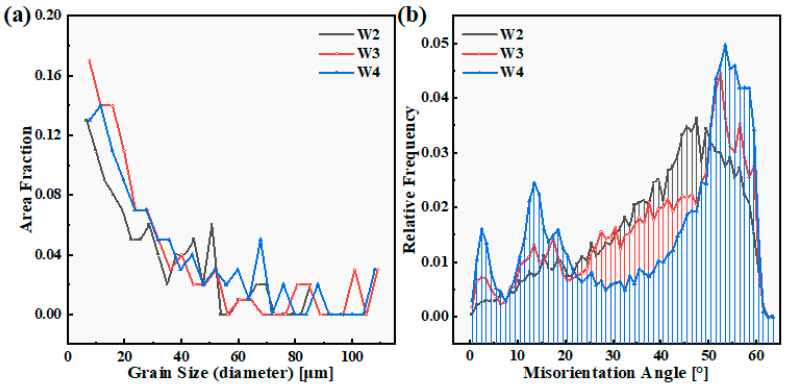
Statistics of the grain size and misorientation angle in the fusion zone of the W2, W3, and W4 welds. (**a**) Grain size (**b**) Misorientation angle.

The statistics of the grain size in the fusion zone of the W2, W3, and W4 welds are shown in Figure 7a. When the welding current was 160 A, there was an unfused area at the bottom of the W1 weld, so the grain size was not considered. The grain size of each weld seam was calculated on the basis of the equivalent circle diameter (unit: μm) of the grains. When the welding current was 170 A, the average grain size in the fusion zone of the W2 weld was 26.906 μm. When the welding current is 180 A, the average grain size in the fusion zone of the W3 weld increases to 29.479 μm. This is due to the increase in the welding current leading to an increase in the welding heat input. The peak temperature of the weld seam during the welding process is high, and some grains further grow into large grains. As the welding current continued to increase, the average grain size of the fusion zone of the W4 weld reached 31.941 μm at a welding current of 190 A. Although there is a difference in the average grain size of the three sets of welds, the difference is relatively small. According to the Hall–Petch relationship, differences in grain size may lead to differences in mechanical properties. The average misorientation angles at the fusion zone positions of the W2, W3, and W4 welds are 39.966°, 39.561°, and 39.287°, respectively. There was no significant difference in the average misorientation angle at the FZ among the three weld seams, but the proportion of large misorientation angles above 50° was highest in W4, followed by W3. This may be related to the increase in welding heat input, resulting in the appearance of large grains.

**Figure 8:**
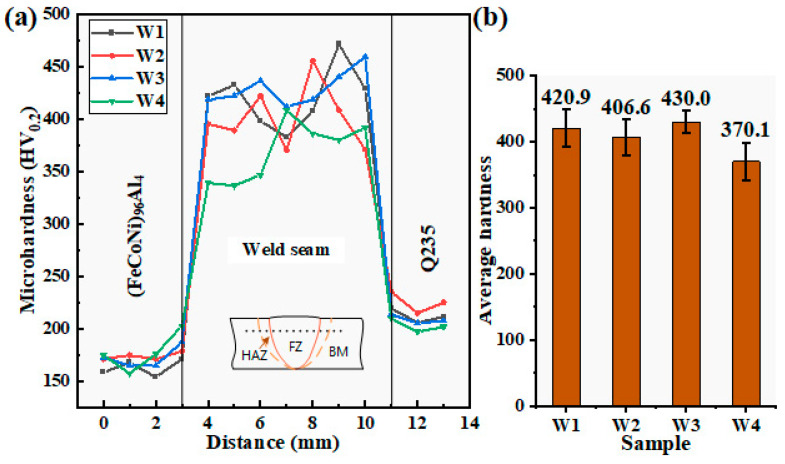
Hardness statistics of the welds and base materials. (**a**) Hardness distribution diagram of the base material and weld seam and (**b**) average hardness diagram of the weld seam.

The hardness measurement results of the weld seam and BM are shown in Figure 8. The microhardness measurement results show that the hardness of commercial Q235 carbon structural steel is between 190 and 220 HV, whereas the hardness of the (FeCoNi)_96_Al_4_ HEA plate is lower than that of the Q235 base material, approximately 160–200 HV, as shown in Figure 8a. The hardness of the weld seam significantly improved, with an average microhardness of over 370 HV. The microhardness of the welded joint is approximately 1.7 times greater than that of the Q235 base material, which is twice the hardness of the (FeCoNi)_96_Al_4_ HEA plate. The increase in hardness of the welded joints may be due to the formation of refined microstructures and needle-like BCC phase structures. When the welding current is 190 A, the average hardness of the joint is the lowest, approximately 370.1 HV. When the welding current is 180 A, the average hardness of the joint reaches 430 HV. The difference in hardness of the joint can be attributed to the large proportion of small grains in weld W3 (with a welding current of 180 A), with an equivalent circular diameter of 30 μ. The proportion of grains within m reaches 70%. With the increasing welding heat input, many grains with large misorientation angles appeared in weld W4. Within the weld seam, the proportion of misorientation angles above 50° reaches 44%. The average hardness values of W1 and W2 are similar, with values of 420.9 HV and 406.6 HV, respectively.

**Figure 9:**
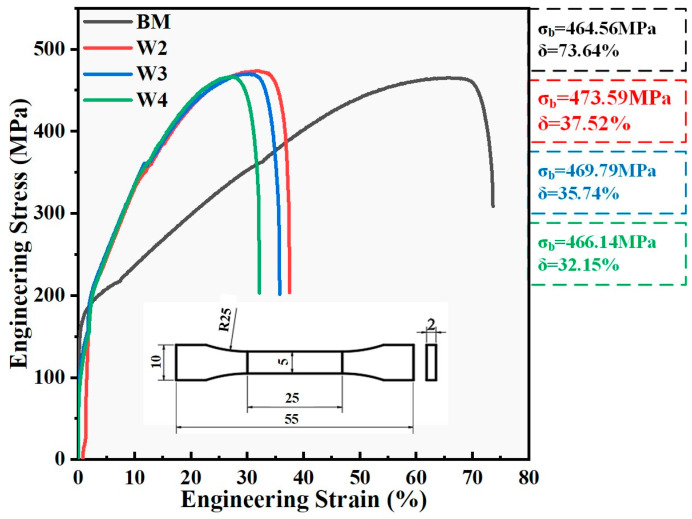
Statistical chart of the tensile test results for the W2, W3, and W4 samples and HEA base metal.

Figure 9 shows the stress–strain curves of the W2, W3, and W4 samples and the (FeCoNi)_96_Al_4_ HEA base material. The dimensions of the tensile sample are shown in the figure. The tensile strength of commercial Q235 structural steel is approximately 400–550 MPa, and the elongation is approximately 24%. The tensile strength of the (FeCoNi)_96_Al_4_ HEA base material is greater than 450 MPa, which is close to that of commercial Q235 structural steel. The elongation is much greater than that of the Q235 base material, reaching over 70%, which means that the plasticity is much greater than that of the commercial Q235 material. The tensile test results revealed that after dissimilar welding of the (FeCoNi)_96_Al_4_ HEA and Q235 steels, the elongation decreased by approximately 50% compared with that of the (FeCoNi)_96_Al_4_ HEA base material but was still greater than the elongation of the Q235 steel while maintaining the strength of the HEA base material. When the welding current is 170 A, the performance of the welded joint is optimal, and the tensile strength of sample W2 reaches 473.59 MPa, with an elongation of 37.52%. As the welding current increases and the welding heat input increases, the performance of the joint slightly decreases, and the elongation of joint W4 is only 32.15%. The tensile test results correspond to the microstructure of the joint. According to the Hall–Petch relationship [30,31,32], the larger the grain size is, the lower the material strength.

**Figure 10:**
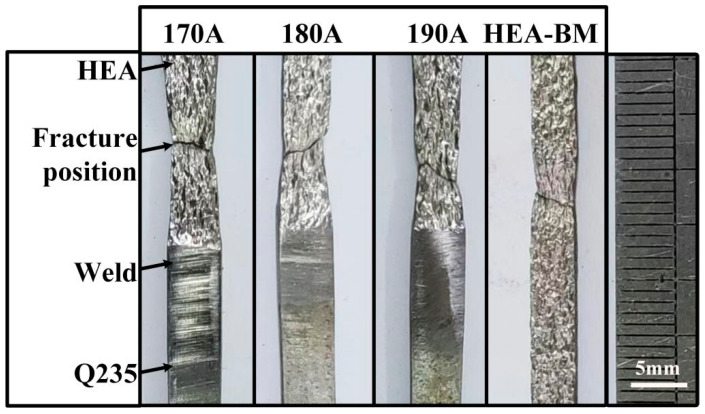
Macromorphology of tensile samples after fracture.

Figure 10 shows the macroscopic morphology of tensile samples subjected to different welding currents and samples of the (FeCoNi)_96_Al_4_ HEA base metal after fracture. After dissimilar welding of the (FeCoNi)_96_Al_4_ HEA and Q235 steel, the samples both fractured near the heat-affected zone on one side of the (FeCoNi)_96_Al_4_ HEA. The postfracture samples of the HEA base material indicate that the overall plastic deformation of the sample occurred during the tensile process. The (FeCoNi)_96_Al_4_ HEA material has good plasticity and can produce significant plastic deformation without fracture under stress. After dissimilar welding of the two materials, the material on the Q235 side showed almost no plastic deformation, whereas the material on the HEA side inherited the good plasticity of the HEA. The dissimilar welded samples subjected to different welding currents all exhibited significant plastic deformation and necking on the HEA side, which is a macroscopic indicator of ductile fracture.

**Figure 11:**
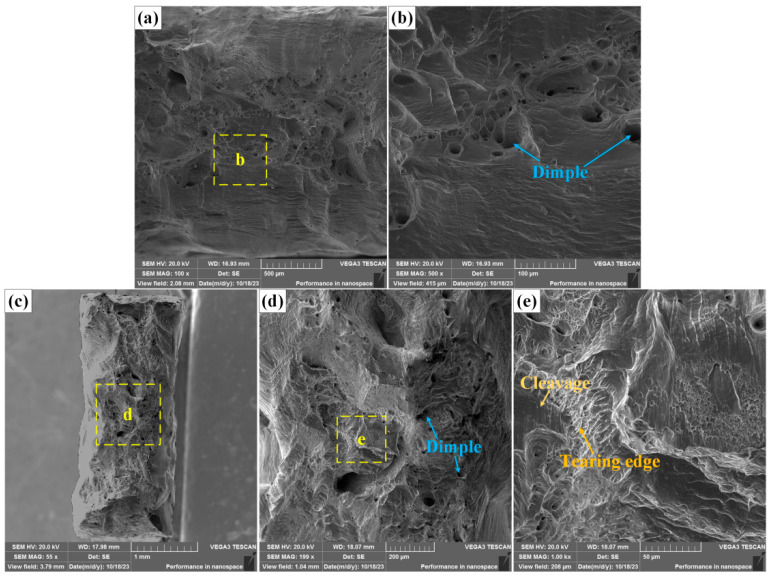
SEM images of fracture surfaces at different magnifications. (**a**,**b**) Fracture surface of the base material tensile sample, (**c**–**e**) fracture surface of the dissimilar joint tensile sample.

SEM images of fracture surfaces with different multiples are shown in Figure 11. The fracture surface of the (FeCoNi)_96_Al_4_ HEA tensile sample clearly has large dimples, with a large proportion of dimples, as shown in Figure 11a,b, which are typical ductile fracture characteristics. After dissimilar welding of the (FeCoNi)_96_Al_4_ HEA and Q235 steel, fractures occurred near the heat-affected zone on one side of the (FeCoNi)_96_Al_4_ HEA. The tensile fracture surface of the dissimilar welded sample still maintains the characteristics of ductile fracture, and ductile dimples and tearing edges are observed in the fracture surface, as shown in Figure 11d,e. However, owing to the influence of the number of welding cycles, the grain morphology changes, and the fracture morphology becomes more complex. In addition to tough dimples and tearing edges, cleavage features have also been discovered. This may be one of the reasons for the decrease in elongation at break. On the basis of the necking phenomenon in Figure 10, it can be inferred that the fracture mode of the dissimilar joint is still dominated by ductile fractures.

## 4. Conclusions

This work focuses on the microstructure and mechanical properties of (FeCoNi)_96_Al_4_ HEA and Q235 structural steel dissimilarly welded samples to expand the application range of Q235 structural steel and promote the engineering application of high-plasticity (FeCoNi)_96_Al_4_ HEAs. The following conclusions are drawn:

When the welding current is above 170 A, the heat input is greater than 557.6 J/mm, and the 3 mm plate is completely melted, resulting in a well-formed welding joint. At the fusion line of (FeCoNi)_96_Al_4_, there is a large area of the heat-affected zone, and a noticeable partial melting zone can also be observed. There is no obvious heat-affected zone boundary near the fusion line of the Q235 steel.

The (FeCoNi)_96_Al_4_ HEA base material has an FCC crystal structure, whereas the fusion zone of the weld seam is almost entirely a BCC structure. The microstructure of the weld seam presents needle-like and block-like grains that are different from those of the base metal. As the welding current increases from 170 A to 190 A, the average grain size of the weld also increases, but the difference in the misorientation angle is relatively small. Owing to the difference in microstructure between the weld seam and the base metal, the average hardness of the welded joint is close to twice that of the (FeCoNi)_96_Al_4_ HEA and approximately 1.7 times that of the Q235 steel.

The tensile test results revealed that the strength of the dissimilar welded sample reached 460 MPa, maintaining the tensile strength of the (FeCoNi)_96_Al_4_ HEA, which is similar to that of the Q235 structural steel. The elongation at break reached over 30%, which was significantly greater than that of the Q235 structural steel. The dissimilar welded samples have a tensile strength close to the base metal and elongation higher than that of the Q235 steel. Its excellent mechanical properties further expand the application range of commercial Q235 structural steel and provide a good foundation for the engineering application of the (FeCoNi)_96_Al_4_ HEA, indicating that the (FeCoNi)_96_Al_4_ HEA can be applied in commercial Q235 structural steel.

## Figures and Tables

**Table 1:** Chemical composition of (FeCoNi)_96_Al_4_ and Q235 steel (wt%).

Material	Fe	Co	Ni	Al	Mn	Si	C	S	P
Q235 steel	Bal	-	-	-	0.33–0.65	≤0.35	≤0.22	≤0.05	≤0.045
(FeCoNi)_96_Al_4_	32	32	32	4	-	-	-	-	-

## Data Availability

The original contributions presented in this study are included in the article. Further inquiries can be directed to the corresponding authors.
